# The Atlas of Living Australia: History, current state and future directions

**DOI:** 10.3897/BDJ.9.e65023

**Published:** 2021-04-21

**Authors:** Lee Belbin, Elycia Wallis, Donald Hobern, Andre Zerger

**Affiliations:** 1 Atlas of Living Australia, CSIRO, Canberra, Australia Atlas of Living Australia, CSIRO Canberra Australia; 2 Atlas of Living Australia, CSIRO, Melbourne, Australia Atlas of Living Australia, CSIRO Melbourne Australia

**Keywords:** Atlas of Living Australia, biodiversity, research data infrastructure, informatics

## Abstract

The Atlas of Living Australia (ALA) is Australia’s national biodiversity database, delivering data and related services to more than 80,000 Australian and international users annually. Established under the Australian Government’s National Collaborative Research Infrastructure Strategy to provide trusted biodiversity data to support the research sector, its utility now extends to government, higher education, non-government organisations and community groups. These partners provide data to the ALA and leverage its data and related services. The ALA has also played an important leadership role internationally in the biodiversity informatics and infrastructure space, both through its partnership with the Global Biodiversity Information Facility and through support for the international Living Atlases programmes which has now delivered 24 instances of ALA software to deliver sovereign biodiversity data capability around the world. This paper begins with a historical overview of the genesis of the ALA from the collections, museums and herbaria community in Australia. It details the biodiversity and related data and services delivered to users with a primary focus on species occurrence records which represent the ALA's primary data type. Finally, the paper explores the ALA's future directions by referencing results from a recently completed national consultation process.

## Introduction

The Atlas of Living Australia (ALA) was established in 2010 by the Australian Government’s National Collaborative Infrastructure Strategy (NCRIS) to support the needs of the Australian and international research community for comprehensive and timely access to Australian biodiversity data. The ALA is now delivering data and related services to more over 80,000 users a year across research, industry, governments and the public. It supports programmes in taxonomy, biodiversity, genomics and ecosystem science, contributes to major natural resource management programmes and supports the international community as the Australian node of the Global Biodiversity Information Facility (GBIF) and the code base for the successful international Living Atlases community. The ALA was established on open-access principles, with data publishers by default using Creative Commons licences and with an open-source code base. This approach has encouraged re-use and maximised the value of data, especially for data that have been funded, produced or collected by public institutions in Australia.

As of February 2021, the ALA holds almost 95 million records associated with more than 111,000 species, predominantly from the Australian region. As a complement to its species data, the ALA also manages a wide range of other categories of data, including information on natural historical collections themselves, spatial layers, indigenous ecological knowledge, taxonomic profiles, biodiversity literature, data on biodiversity projects and animal tracking data. Investment in the ALA and in its partner capabilities (including GBIF and the Living Atlases) has radically enhanced ease of access to biodiversity data.

Fundamental to ALA's business has also been the development of tools and platforms to enable different stakeholders to collect, manage and deliver open biodiversity data. Examples include BioCollect for field-based data collection and management, DigiVol to engage volunteers in the digitisation of analogue data, the Australian node of the Biodiversity Heritage Library, ALA's spatial portal and species lists tools. Most recently, the ALA has partnered with the global iNaturalist platform to support citizen scientists in the acquisition and identification of biodiversity observations. Collectively, this portfolio of capability has been fundamental in improving how ALA captures and utilises biodiversity data.

This paper provides a history of the ALA including its origins and key drivers, a description of the data and services it delivers and concludes with a summary of the findings from recent stakeholder consultations that will provide information for the ALA’s future directions. Although these consultations focused on the ALA, the results offer insights of importance to other national and international biodiversity data infrastructures regarding future trends, stakeholder expectations and limitations around current approaches to delivering biodiversity data and services.

## Biodiversity in Australia

As a result of its isolation for around 100 million years and its distinctive environment, Australia's fauna and flora are rich and unique, exhibiting high degrees of endemism. Human influence has led to significant loss and/or transformation of this biodiversity (Woinarski et al. 2019). Environmental challenges and human consumption place unprecedented and increasing stresses on the environment and species. At the global scale, the Intergovernmental Science-Policy Platform on Biodiversity and Ecosystem Services (IPBES 2019) reports rapid losses in biodiversity and ecosystem health, and states that insufficient information exists to monitor and respond to these trends. Biodiversity researchers and managers commonly face the challenge of delivering and interpreting disparate information to answer the greatest environmental questions facing society. For example:

- Signs point to massive losses in insect numbers and diversity, but what is the actual scale of these losses and the implications for ecosystem health? (Braby 2018)
- Australia is home to a number of global biodiversity hotspots, which have preserved unique evolutionary lines through many climatic changes. How are current and expected pressures on these ecosystems likely to modify these areas?
- How can we design effective ecosystem restoration programmes and respond to ecological changes in response to major disturbances, such as the 2019–2020 bushfire season?

The Australian Government’s recent Independent Review of the Environmental Protection and Biodiversity Conservation Act Interim Report (Samuel 2020) identified that a ‘quantum shift in the quality of information, accessible data and information available to decision-makers’ is necessary to support future regulatory environmental management programmes. The ALA has an important role to play in supporting these emerging regulatory policy needs of government.

Historical information on species distributions and population abundances is central to ecology, conservation and to all areas of environmental planning and sustainability. The ALA is highly regarded for the progress it has made over the last 10 years in significantly improving open access to integrated information about species from previously diverse and isolated sources. This impact has been valued by both national and global research communities.

## Establishment of the ALA

Australian herbaria and museums have a long history tackling the issues of data sharing, standards and collaboration for natural history specimens. Standards development for this work in Australia took place in an international context through involvement of Australian biologists and data scientists in Biodiversity Information Standards (known by the acronym TDWG) from its inception in the mid-1980s (http://old.tdwg.org/about-tdwg/history/). The botanical community first published the HISPID data standard for herbarium specimens in 1989 (http://www.anbg.gov.au/projects/hispid/hispid3.html). In the late 1990s, the peak body for herbaria in the region, the Council of Heads of Australasian Herbaria, formed a consortium with the Australian Biological Resources Study (ABRS, https://www.environment.gov.au/science/abrs), which held responsibility for coordination of taxonomic research. This consortium sought funding to digitise herbarium specimens in all the state and federal herbarium collections and received AUD$10 million for this purpose from the Australian Government, states and territories and private sources. Its focus was on the capture of herbarium specimen data into electronic databases, with the eventual goal of producing an online resource. The result was Australia’s Virtual Herbarium (now Australasian Virtual Herbarium, AVH, established in 2001). AVH currently provides access to eight million records for specimens of plants, bryophytes and fungi across Australia and New Zealand, both directly and through its connections to the ALA (https://avh.ala.org.au).

Internationally, interest in establishment of biodiversity data infrastructures had been growing for more than a decade before the ALA was established. The Biodiversity in World Science Report, published by UNESCO in 1996, identified biodiversity as ‘our most precious “unknown”’ and made the case for developing better understanding of genes, species and ecosystems (di Castri 1996). In Australia, the Environmental Resources Information Network (ERIN, https://www.environment.gov.au/about-us/environmental-information-data/erin) was launched in 1992 through the former Australian Government Department of the Environment with the first national remit to draw together, supplement and make publicly available biodiversity data from the environmental departments from Australian states and territories.

By 1999, the OECD had developed a focus on research infrastructure and emphasised the need for international collaboration. One result was the recommendation by the OECD Megascience Forum Working Group on Biological Informatics in 1999 (OECD Megascience Forum 1999) to establish the Global Biodiversity Information Facility (GBIF, https://gbif.org) to make biodiversity data and information accessible worldwide. GBIF was conceived as an online network funded by membership fees from participating countries, who would contribute data to GBIF through their national ‘nodes’. The Working Group’s recommendations were shaped by the experiences of a handful of key countries in developing systems, based especially on digitised botanical specimens, including Australia through ERIN.

In the early 2000s, the zoological community in Australian museums formed a peak body called the Council of Heads of Australian Faunal Collections (CHAFC). This group was also interested in sharing data through a public website. The zoological community had to engage in extensive discussions to overcome philosophical hurdles around open data sharing. In particular, the community was concerned that providing precise locality data for threatened and rare species would encourage poaching and illegal collecting. There were also concerns about protecting the privacy of collectors and donors. Technology fixes were proposed for data sensitivity issues, including denaturing locality data through gridding and excluding some data elements from sharing arrangements. The result was OZCAM – the Online Zoological Collections of Australian Museums, now a portal in the ALA (https://ozcam.ala.org.au). The establishment of the ALA owes much to the prior existence of AVH and OZCAM.

The Australian Government commissioned a review of national research infrastructure with the intention of funding new initiatives. The resulting National Collaborative Research Infrastructure Strategy Roadmap in 2006 (Department of Education 2006) outlined 16 areas of priority infrastructure, including Integrated Biological Systems. The Government sought a proposal from the collections community to continue their existing efforts to database animal, plant and microbial collections and aggregate the results into a single online platform. A group of approximately 25 representatives from Australia’s biological collections, with representatives from ABRS, AVH and OZCAM, met at the Australian Museum in Sydney in May 2006. This meeting identified many benefits from an aggregated database, based on existing standards and settled on the concept of the ‘Atlas of Living Australia’. The collections developed a strong case that the data held by collecting institutions should be considered as significant research infrastructure. A submission from the major museums and herbaria was successful and CSIRO was appointed as the contracting agency.

## The original scope of the ALA

The ALA was approved for Australian Commonwealth funding as part of the NCRIS programme starting in 2007. NCRIS established a new generation of Australian national research infrastructures (NRIs) to promote and support world-class research across multiple domains. The rationale for the ALA funding was to enhance access to Australia’s biological collections as an ‘important supporting infrastructure for research relating to models of disease, biosecurity and biodiversity, and supporting quarantine, environmental remediation and management.’ Accordingly, the ALA was established as a partnership between CSIRO (which curates national collections for multiple taxonomic groups), major state and territory museums and herbaria (and the associated national Councils), key university collections, the Australian Biological Resources Study (ABRS, responsible for the national species lists and funding for taxonomic projects) and the pest collections of the Department of Agriculture, Forestry and Fisheries and the Victoria Department of Primary Industries.

The announcement in 2007 also created two other NRIs with relevance to biodiversity and the wider work of the ALA: The Integrated Marine Observing System (IMOS: http://imos.org.au/) and the Terrestrial Ecosystem Research Network (TERN: https://www.tern.org.au/). IMOS and TERN were established to support environmental research and data management in the marine and terrestrial spaces. In both cases, the scope included data collection and processing activities that relate to biodiversity composition across space and time. The original NCRIS strategy did not specify how linkages would be formed between ecological datasets and the largely collection-based data of the ALA.

The ALA itself was positioned in an Integrated Biological Systems cluster with the Australian Plant Phenomics Facility (APPF: https://www.plantphenomics.org.au/) and the Australian Phenomics Network (APN: http://australianphenomics.org.au/). This association was based on all three NRIs delivering integrated data related to target species, and the ALA was initially given responsibility to provide informatics support for APPF and APN. The Australian Biosecurity Intelligence Network (ABIN) was also funded as an NRI to address requirements around all aspects of biosecurity and overlapped with the ALA in the area of observations and collections of pest species. Other investments with scope relevant to the ALA included Bioplatforms Australia (BPA: https://bioplatforms.com/) for "-omics" technologies and the Australian Urban Research Infrastructure Network (AURIN: https://aurin.org.au/) which addresses issues around urban environments.

Simultaneously with the establishment of these NRIs, a set of cross-domain research data and computing infrastructures were created as the NCRIS Platforms for Collaboration cluster. These included the Australian National Data Service (ANDS) to address issues around data management and storage, and the Australian Research Collaboration Service (ARCS) to support collaborative activities. These two facilities have now merged and evolved into the Australian Research Data Commons (ARDC: https://ardc.edu.au/).

All NRIs, apart from ABIN, are still active and have developed in parallel with the ALA. The Australian Government’s approach through NCRIS has been transformational in encouraging collaborative effort throughout the Australian research sector and in delivering a wide range of datasets and tools for use by researchers and the wider community.

Some downsides resulted from the simultaneous establishment of all NCRIS infrastructures. The ALA needed to solve many issues in research data management long before Platforms for Collaboration could offer stable and standardised models. This forced the ALA to develop its own approaches to metadata standards, vocabulary services, repository services and GIS functionality. Some of these elements are currently addressed in more standardised ways by ARDC and other infrastructure partners. Similarly, the ALA, TERN and IMOS all had to address their own needs around biodiversity data management before reaching the necessary maturity to interconnect services. The overlap in responsibilities between the ALA and ABIN limited opportunities for the ALA to fully address the issues associated with biosecurity collections. Linkages with APPF and APN absorbed some ALA resources at an early stage, but the three infrastructures shared no use cases and these relationships have weakened over time. If these initiatives had been in a more mature state when the ALA was brought into existence, it would have significantly affected how decisions were made about priorities and investments.

NCRIS funding (AUD$8.5 million over the period 2006–2011) gave the ALA the stability to address the following initial scope, using digital content from the partner institutions:

- names and nomenclatural data
- specimen and observational data
- descriptions and descriptive data
- DNA and genetic data
- multimedia

The role of the ALA, as established under NCRIS, was to build the data integration infrastructure required to support research use of the natural history collections and associated digital assets. It was not funded for the generation of new digital content.

In May 2009, as part of a national response to the Global Financial Crisis, the Australian Government announced the Super Science Initiative, a $989 million initiative to build and create research infrastructure, funded through the Education Investment Fund (EIF). As part of the Super Science Initiative, the ALA received an additional AUD$30M to build on and enhance its work through the period 2009–2011. The scale of the funding and the short time period justified a significant expansion of the scope of the ALA’s work across five major areas:

- Collection Data Management – tools and services to optimise the data supply chain through Australia’s natural history collections, from field collection through accession, digitisation and web publication. This included support to reinforce tools around key national collection platforms, including AVH and OZCAM.
- Rich Data Stores – shared infrastructure to manage and maintain biodiversity datasets on behalf of Australian institutions and projects, including mirrors or local nodes for the Biodiversity Heritage Library (BHL: https://www.biodiversitylibrary.org/), Morphbank (https://www.morphbank.net/) and the Barcode of Life Database (BOLD: http://www.boldsystems.org/) and upgrades to the DELTA software for taxonomic identification keys (https://www.delta-intkey.com/).
- Australian National Species Lists – tools, services and expert curation to bring together and complete the Australian National Species Lists, including the Australian Plant Name Index (APNI: https://www.anbg.gov.au/apni/), the Australian Plant Census (APC: https://www.anbg.gov.au/chah/apc/index.html) and the Australian Faunal Directory (AFD: https://biodiversity.org.au/afd/home), together forming the taxonomic framework for Australian biodiversity data.
- Spatial Data Management – shared models, tools and services to ensure interoperability of all spatial data accessed through the ALA and compatibility with data shared through related NCRIS capabilities (particularly TERN and IMOS). This activity led to the development of the ALA’s Spatial Portal (Belbin 2011, https://spatial.ala.org.au).
- Data Dissemination – web portals and applications to improve delivery of biodiversity data to end-user communities, including conservation and biosecurity stakeholders and citizen scientists.

Some of these areas had limited long-term impact, but the EIF funding established the scope still delivered by current ALA data and services. Most significantly, the work on the Australian National Species Lists represented a recognition within NCRIS that core datasets such as these can themselves be regarded as significant national infrastructure. This recognition allowed funding to be directed to taxonomists for contributions to expand the coverage or quality of sections of the national species lists.

The ALA was conscious from its inception of the significant role that citizen science could play in contributing valuable information to Australia’s biodiversity. This undertaking has evolved into ALA projects and capabilities, such as DigiVol and BioCollect, covered below.

## ALA data and services

**Figure 1.**
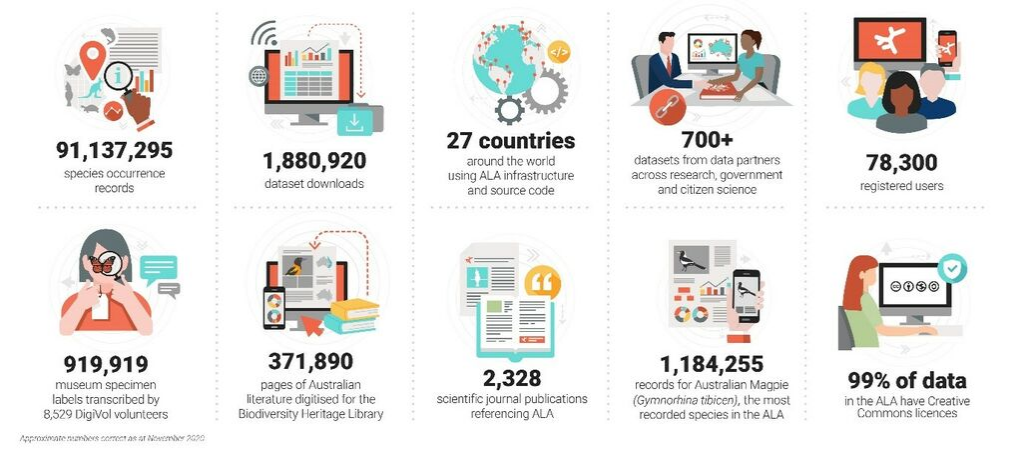
Summary metrics describing dimensions of the ALA. Real-time data regarding selected metrics is also available at https://dashboard.ala.org.au/.

As of January 2021, the ALA contains nearly 95 million occurrence records of over 111,000 species from a total of over 195,000 species listed in the Australian National Species Lists (https://www.rbg.vic.gov.au/science/projects/taxonomy/atlas-of-living-australia-national-species-lists-project). A total of 84.4% of the records are terrestrial and relate to areas of Australian jurisdiction, 8.8% are marine, while the remaining 6.8% of the records are spread across 270 other countries and dependencies. The ALA contains data from observations of Australian species outside the Australian region and also data on introduced species that can be found in the Australian region. Of the total records, around 72 million are field observations (categorised as human observations in the Darwin Core standard: Wieczorek et al. 2012) and 13.5 million are of preserved specimens. There are 1.7 million machine observation records and we anticipate these will rise as a proportion of total records. The earliest Australian record is from the late 1600s and, on average, thousands of occurrence records are being added daily. Fig. 1 provides an overview of summary metrics describing the various dimensions of the ALA.

Field observations of species range from single *ad hoc* sighting records to hundreds of data collections from over 500 institutions that provide data to the ALA. The largest single data provider is Birdlife Australia (https://birdlife.org.au/) with over 15 million records. As noted elsewhere in this paper, the ALA also manages bio-related terrestrial and marine environmental layers, species lists, images, sounds, ecological related projects and over 500,000 location definitions in its gazetteer.

Over 800,000 specimen labels and 124,000 pages of field notes have been transcribed by over 6000 public volunteers using the ALA’s DigiVol volunteer portal, hosted by the Australian Museum (http://digivol.ala.org.au/). Collecting institutions around the world have an incredible backlog of specimens, images, field notes and archives that are inaccessible because they have not yet been digitised. DigiVol provides a way to harness the power and passion of volunteers to help in the digitisation effort to make more information available to science. Recently, an additional area that can benefit from volunteer input is around automated camera trap data where the task is to identify and tag animals in photographs taken by cameras mounted in the environment. Along with its success at attracting volunteers, DigiVol is an excellent example of making infrastructure meet many different objectives.

**Figure 2.**
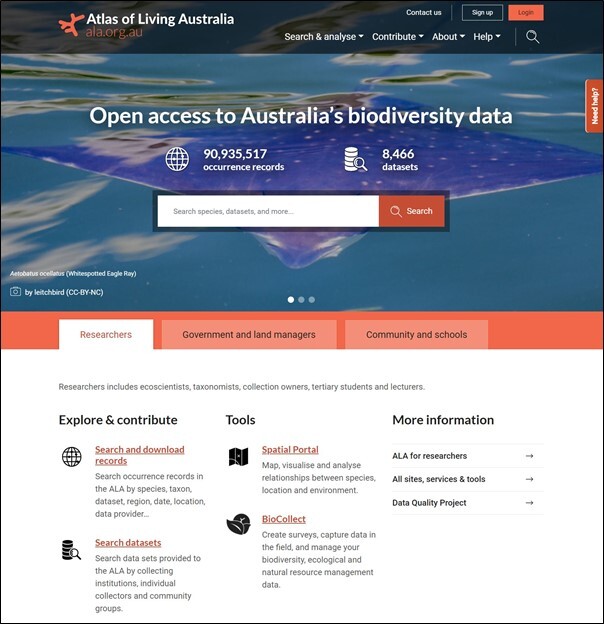
ALA landing page at https://ala.org.au/

The wide range of data types requires the ALA to maintain an equally wide range of services to accept, process and expose the data to meet the needs of diverse communities. The landing page of the ALA (Fig. 2, https://ala.org.au/) provides a simple search for species, datasets and most of the information in the ALA. It is possible to explore species-level information and drill down to any of the occurrence records. A suite of application programming interfaces (APIs) are provided, as well as CSV downloads and an ALA4R environment to support further research using data from the ALA, but outside of the ALA website. The ALA also maintains a suite of portals that support specific communities or specialised data and services, each with a web address of the form <portalname>.ala.org.au. Examples include https://dashboard.ala.org.au/ – a data dashboard listing dimensions of ALA data holdings and usage; https://biocollect.ala.org.au/ for ecological project management and data collection; https://spatial.ala.org.au/ – a map- and analysis-focused portal for the research community; and https://lists.ala.org.au/ for lists that group species for any purpose, including threat categories, presence in defined areas or common traits. A more complete list of portals and services can be found at https://www.ala.org.au/sites-and-services/.

The ALA's Biocache (https://biocache.ala.org.au) is a tool that provides an organised view combining and linking specimen data, genetic information, field observations, sampling events, animal tracking data and media collected by diverse stakeholders and arranges these data for search, access, analyses and download through a standardised record structure. The Biocache includes records from museum and herbarium specimens, citizen science observations, field surveys, eDNA studies, literature, remote sensing, electronic tags and machine observations and any other biological research activity that records the occurrence of species in time and space.

The ALA is active across three of the tiers for biodiversity informatics identified in the Global Biodiversity Informatics Outlook (GBIO, Hobern et al. 2012), promoting common and consistent approaches around FAIR (findable, accessible, interoperable and reusable) and open access to biodiversity data (Culture tier), supporting biodiversity stakeholders in turning their assets, observations and measurements into digital formats (Data tier) and developing the integrated views required by users to make use of these data (Evidence tier). The ALA supports work in the fourth tier (Understanding tier) by making all these views and services accessible for researchers and decision-makers to apply in their work. FAIR data and software code underpin all ALA products and services.

**Figure 3.**
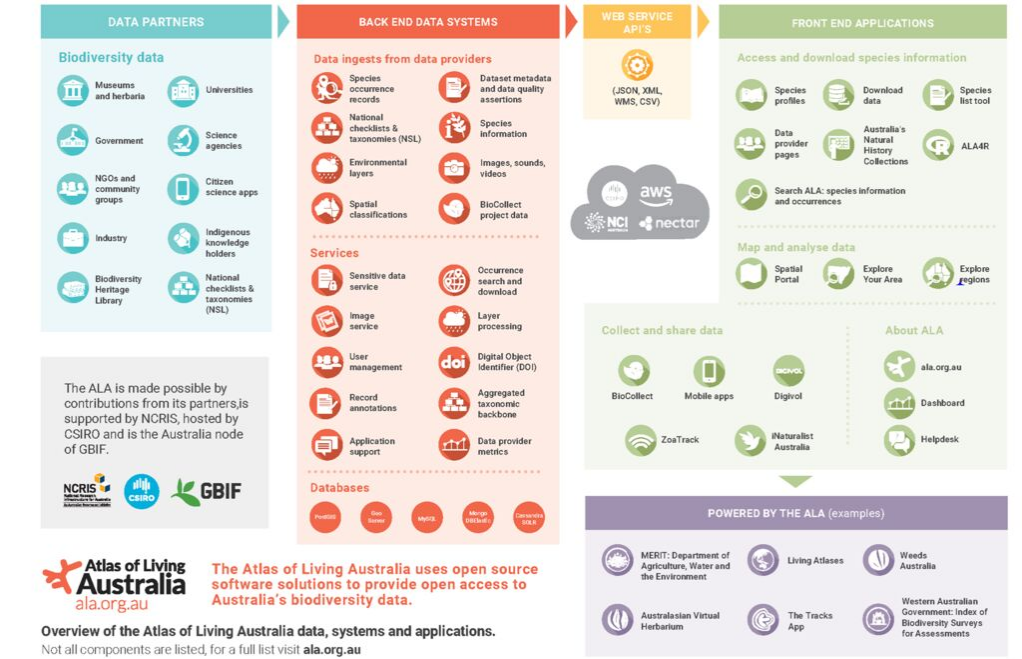
Overview of ALA data partners, data systems and user applications.

The following sections provide a summary of ALA activities by data type. Each section provides an outline of the nature of the data, how it is processed and how the data are exposed publicly through various portals and tools. Fig. 3 provides a schematic overview of the relationship between data partners, data systems and applications to support ALA users and Table 1 provides URLs for key data described under Sections 4.2 to 4.8.

### Taxonomic framework

The most fundamental axes for organising biodiversity data are taxonomy (species identifications), space (coordinates) and time (dates and timestamps). The taxonomic dimension is the most complex and difficult to standardise of these three and normally relies on the accuracy and precision of scientific names supplied within data. Scientific names are applied to occurrence records, species lists, species information and most other data types within the ALA. The ALA handles these names through an integrated classification with accepted names and synonyms for each species and uses this to index all data with a taxonomic name component.

The ALA does not assume authority for determining what native and non-native species occur within the Australian region or what names should be preferred for these species. It relies instead on the canonical sources of taxonomic name lists for Australian species (and New Zealand species, given the Australasian focus of many users), including those managed as the Australian National Species Lists:

- the Australian Faunal Directory for all animal groups (https://www.environment.gov.au/science/abrs/online-resources/fauna)
- the Australian Plant Names Index and the Australian Plant Census for vascular plants (https://biodiversity.org.au/nsl/services/)
- AusFungi for fungi (see https://www.rbg.vic.gov.au/science/projects/mycology/catalogue-of-australian-fungi)
- AusMoss for mosses (https://moss.biodiversity.org.au/nsl/services/AusMoss)
- the New Zealand Organisms Register for all New Zealand organisms (http://www.nzor.org.nz/)
- the Catalogue of Life (http://www.catalogueoflife.org/) for cases where no match can be found to the name supplied or where the occurrence record is for a species not found in Australia
- the World Register of Marine Species (http://www.marinespecies.org/), which is not currently used by the ALA, but will be integrated in the future.

Where possible, the ALA links from species pages to literature in the Biodiversity Heritage Library and also in the National Library of Australia’s Trove (https://trove.nla.gov.au/). The ALA taxonomic framework includes a number of services that are heavily used across the ALA infrastructure and also exposed for use by external users. The primary function is name-matching to determine how to interpret scientific name strings. When an occurrence record is ingested into the ALA, it may include a currently accepted scientific name or a synonym that is no longer in use or depends on an alternative classification. A particularly important case is where a name may no longer be accepted in the light of current taxonomic knowledge, but has been written into legislation, for example, to refer to threatened species. Such uses must be interpreted correctly.

The ALA name-matching service seeks to determine which taxon is intended by each name included in a data record. When the name is included in an authoritative checklist source, either as an accepted name or as a known synonym for a recognised taxon, processing is simple. Synonyms can be mapped as references to an accepted taxon, allowing the occurrence record or other information to be correctly handled in the context of current taxonomic understanding. Processing may be more complex in the case of names that have historically been misapplied or have been used to refer to a broad species concept that is currently treated as several accepted species (pro-parte synonyms).

The service must also resolve taxonomic names even if there is no exact match in the index, possibly as a result of spelling errors. In such cases, the ALA uses a fuzzy-matching algorithm to seek a ‘closest’ match, but this process may return false positives. Alternatively, the ALA can handle difficult cases by ‘upmatching’ the name to a higher-ranked taxon – for example, mapping an occurrence record that was supplied as a species record with a binomial scientific name to the genus or family level or even higher. Mesibov (Mesibov 2013, Mesibov 2018) provides an analysis of the name-matching process, reporting that up to one in five supplied names may be changed, a process that he concludes, in some cases, loses or confuses data. When the ALA matches a name, it stores both the provided name and the name selected as the best match. This ensures that the original data are preserved and users can freely re-interpret them.

The name matching service not only attempts algorithmically to find the best match for any scientific name, but also fills out the higher classification and flags possible data issues. This process relies on the quality of the canonical sources available to the ALA and on algorithms developed to apply the information they contain. Since the process is automated, errors may occur particularly for names not recorded in these sources or for names that are misspelled or malformed. The code and documentation for the ALA name matching process is available from GitHub:

- the name-matching algorithm: https://github.com/AtlasOfLivingAustralia/ala-name-matching/blob/master/doc/matching-algorithm-v2.md
- the Biocache-store processing code: https://github.com/AtlasOfLivingAustralia/biocache-store/blob/master/src/main/scala/au/org/ala/biocache/processor/ClassificationProcessor.scala
- descriptive information about the conventions used to assemble the names index: https://github.com/AtlasOfLivingAustralia/bie-index/blob/master/doc/nameology/index.md.

### Species occurrence data

Most data shared with the ALA are treated as occurrence records, broken down as follows:

- Over 80% of occurrence records are from field observations collected either as individual observations (for example, by citizen scientist naturalists) or during systematic surveys of geographic areas or organisms. A proportion of these records are supported by images, video or sounds, but many are not directly associated with any evidence that can be revisited to confirm the observation. Over time, the proportion of unevidenced records is expected to decline as the ALA partners with iNaturalist and other citizen science platforms that encourage and facilitate easy sharing of images or sound recordings.
- Approximately 15% of occurrence records are for preserved specimens held by museums, herbaria and other institutional collections. The evidence to support the data for these records is the specimen on the shelves within the collections.
- Currently approximately 1.7% of occurrence records come from ‘genetic’ studies – including sequence data, tissue data and environmental DNA. This figure is destined to rise quickly in the future.
- A currently small proportion of records derives from animal tracking datasets.

Data quality is one of the most significant aspects to understand when using aggregated occurrence records. ‘Data quality’ is, however, a complex term that will mean different things to different users (see Belbin et al. 2013). In some cases, data are considered poor in quality because they contain errors – for example, mistakes in species identification or metadata. In an aggregation of over 90 million records from diverse sources, it is inevitable that there will be errors. Even records with such failings may be valuable for some use cases. From a user perspective, data quality may be judged in terms of ‘fitness for use’ (see Chapman et al. 2020). The concept is that a record that is unsuitable for one application may be suitable for another. The ALA performs around 100 standard data quality tests on all occurrence records belonging to any of the categories listed above. These tests are designed to flag errors or unlikely values in the data and are largely independent of the nature of any evidence associated with the record. These are stored as assertions that the data provider may review and may be able to fix. Flagging potential issues assists downstream users in reviewing data for their applications. The data quality tests are automatic. Owing to the volume of data entering the ALA and the shortage of taxonomic specialist expertise, there is no manual check of the records or the results of the testing.

The ALA has long recognised the importance of data quality and commenced a project in 2020, both to help data providers detect issues that can be fixed and to help users evaluate data fitness for use. User evaluation of occurrence records relies on the use of filters. Users typically begin by searching for taxa of interest and then accessing the occurrence records relating to that taxon. They may then derive a subset of the records using filters that narrow selection using supplied values for Darwin Core terms, processed values that had been applied to the record, assertions from the ALA tests, various options, based on descriptions or status of a species (sensitive, endangered, invasive etc.) and location-based additions derived by intersecting the location of the occurrence records (where they have a dwc:decimalLatitude and dwc:decimalLongitude) with all environmental data layers held in the ALA (see below). One of the ALA tests checks to see if the record is an outlier on one or more of five selected terrestrial environmental layers, as compared with all records of that taxon. This is a typical example of a test that will ‘flag’ a warning for the record but that, without additional information (which may not exist), does not guarantee that the record is invalid. The record may, for example, be a species that is adapting to climate change by extending its range south. The ALA, GBIF and iDigBio have agreed to implement the work of Biodiversity Information Standards Task Group on a core set of ‘Tests and Assertions’ (https://github.com/tdwg/bdq/tree/master/tg2).

While useful automated tests can be written to assess the location and time aspects of an occurrence record, this is not feasible for the identification (species name). For observation records, particularly *ad hoc* records, the ALA and similar projects rely increasingly heavily on peer or community review. Capabilities such as iNaturalist (https://www.inaturalist.org/places/australia) engage a volunteer pool of international expertise to validate the identification of species observations. Before 2019, the ALA had several mobile applications that offered no peer review, as well as BowerBird, an observation platform hosted by Museum Victoria, that did provide peer review. In 2019, the ALA became a member of the iNaturalist Network and established an Australian iNaturalist node to encourage observers to submit their species observations using a tool that would provide an improved level of validation. Records were transferred from BowerBird to iNaturalist with the assistance of the BowerBird community.

The ALA preserves raw data as supplied for occurrence records. The general principle adopted in the ALA is that no original data should be overwritten – a concept that is also captured in the ‘verbatim’ terms of Darwin Core, for example, ‘dwc:verbatimCoordinates’ and ‘dwc:verbatimeventDate’. No data are overwritten without documenting the actions and justification. There is, however, no current standard that captures everything that may happen between the raw data submission and a final processed data product. The ALA encourages any registered user to annotate potential issues associated with an occurrence record.

All species occurrence records can be accessed through the ALA’s home page and several other ALA portals. From the home page, the primary search returns matching species and higher taxa, common names and other data resources, such as spatial layers, datasets and collections. Each taxon overview page displays images, a map of the spatial distribution (with links to the Spatial Portal), a link to a list of records, any conservation status, a description and references (see Species Information below). From a listing of records, any number of filters can be applied. Records can be summarised by charts and downloaded with any combination of selected terms and ‘value-added’ terms in CSV format.

The ALA also provides over 100 Application Programming Interface (API) endpoints to access species occurrence data and services (https://api.ala.org.au). These functions enable URLs to be used from a browser or programming language to return results tuned to user requirements. The API is grouped into functions for occurrences, species level information, lists, datasets, species names, volunteer portal, environmental layers, data dashboard and regions. API responses may be returned in multiple formats, including web pages, images, ZIP files, shapefiles, and CSV, JSON, RDF, KML, WKT and EML files. The ALA website and other front-end systems also utilise these APIs. ALA4R (https://github.com/AtlasOfLivingAustralia/ALA4R) is an alternative programmatic access route to ALA data and services for those who prefer to use the R environment (https://www.r-project.org/) for data analyses.

**Figure 4.**
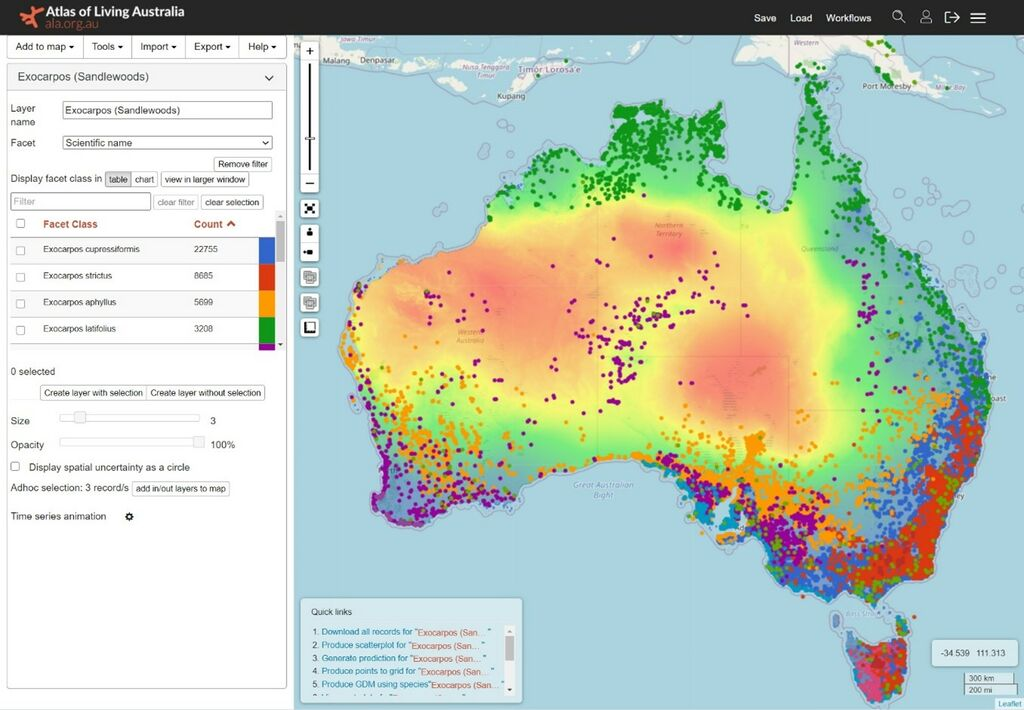
The Spatial Portal. Distribution of genus *Exocarpos* in the family Santalaceae (sandalwoods) with mean annual evaporation as a background. To reproduce: https://spatial.ala.org.au?ss=1601846523658.

The Spatial Portal (Fig. 4) is the ALA’s primary portal to service the research community, environmental managers, environmental consultants and enthusiastic citizen scientists. As its name suggests, the portal has a spatial emphasis and includes a cross-section of analytical tools. Users can enter the portal, search for species or an existing species list (see below: https://lists.ala.org.au), use an advanced filtering tool, visualise and analyse records. An alternative strategy available in the Biocache and Spatial Portal is to identify occurrence records based on any combination of occurrence record terms and ALA-added terms. For example, it is possible to list records of bird species that have associated images, a CC0 licence and coordinates that do not match the supplied state or territory.

The ALA also permits area-oriented searches for occurrence records. Users can explore biodiversity within user-defined areas using the Explore Your Area tool (https://www.ala.org.au/explore-by-location/) or within pre-defined regions and areas using the Region tool (https://regions.ala.org.au) or the Spatial Portal (https://spatial.ala.org.au). The Spatial Portal provides 16 options for how areas can be defined and selected, from clicking on the map, searching the gazetteer, importing or using an environmental envelope. This area-centric approach enables users to answer a basic question: ‘What biodiversity-related things (species, projects, literature etc.) occur in this area?’ The Spatial Portal’s area reports provide a breakdown of species and records by life form, conservation or invasive status, and allow a user to list and download the resulting species lists or occurrence records.

#### Specimen data and genetic materials

Data related to preserved specimens are provided by museums, herbaria, national collections and universities (Figure 4). Preserved specimens are a primary source of high-value data since specimens can be examined repeatedly, yielding new data each time. The ALA receives a subset of the data held by collection institutions, extracted from their institutional collection management systems and mapped to Darwin Core terms. Coverage is from most Australian collections and (via GBIF) from selected international collections.

Museums and herbaria use a range of commercial, open-source or in-house collection management systems: Specify, EMu by Axiell, and Vernon Systems are in use in Australian institutions. The ALA receives collections data in a variety of ways – commonly by having the data packaged as Darwin Core Archives and less commonly by harvesting directly from these systems. The ALA is looking to implement the GBIF Integrated Publishing Toolkit more widely to help streamline repeated data provision from collections.

Emerging data types based on specimens include 3D images, using techniques such as CT scanning, photogrammetry and stacked microscope imagery. A challenge for the ALA will be to allow these new image types to be linked to the occurrence records and viewed (University of Cincinnati 2019).

Specimens from collecting institutions provide fewer records, but cover a greater breadth of species, than field observation records. Researchers working in the collections sector include many taxonomists, whose professional skills are in describing the diversity of the natural world. Collections also extend back several hundred years and include species that are now threatened or extinct. Taxonomists and collections specialists also deliberately collect organisms from every environment to document diversity. ALA records for deep-sea marine invertebrates, cave-dwelling stygofauna, algae and other ‘non-charismatic’ organisms have, for the most part, been supplied from specimens in collections. Interestingly, records for some exceedingly common species also mainly come from collecting institutions – for example, for abundant but very small or cryptic insects and for grasses. The ALA only has 980 occurrence records for the common mosquito, *Aedes notoscriptus*, 770 of which are from preserved specimens. The relative paucity of observations of house flies (*Musca domestica*), pigeons (*Columba livia domestica*) or dandelions (*Taraxacum officinale*) is likely to be both because of their ubiquity in the environment and because observers under-record introduced and pest species.

Genetic data and genomics research are increasingly important in the study of biodiversity. Advances in genetic techniques mean that DNA can now be extracted even from very old museum specimens and this supports new research into evolution and phylogeny. The ALA has not yet found an ideal way to incorporate genetics data into its systems. So far, collecting institutions have started to supply data associated with tissues (e.g. muscle, heart, skin) that have been specifically preserved at the time of collection to permit future genetics work to be conducted. The ALA holds approximately 180,000 tissue records, with data structured according to a relatively new standard, the Global Genome Biodiversity Network (https://terms.tdwg.org/wiki/GGBN_Data_Standard). An even newer type of data that ALA and GBIF alike are now needing to consider are data derived from environmental DNA (eDNA). Following a project that tested how eDNA could be ingested as occurrence records, there are now 1.17 million such records in the ALA.

#### Field observation data

A field observation of a species is documented through, but far from limited to, three primary dimensions: a name (at species or high taxon level), a location and a date/time. With these three components, many questions can be answered. For example, ‘Where has *Eucalyptus gunnii* been observed?’, ‘Were there any observations of *Alaba vibex* after 1960?’ and ‘What reptile species have been observed in the Kimberley region between the years 1950 and 2000?’ The precision and confidence associated with each of these three elements varies. An organism may only have been identified to genus, or may be attributable to a local variety. The location may have been measured with high precision (e.g. one metre resolution) or derived from a general text description. Some observations will be timestamped to a day, while others may only indicate a year. No minimum information standard is imposed for these properties, since imprecise data may remain valuable for users interested in other aspects of the record. In all cases, the values included for each element may be incorrect or imprecise as a result of human or machine error during observation, identification, data entry and data processing. Field observations of species do not have a specimen that can be referred to, but they may contain links to images, audio or video that provides evidence of name, location and date.

Each species observation in the ALA has a dual purpose. First, it indicates that evidence exists for the recorded occurrence of the given species at the given location within a specified time period. Second, where applicable, observation records can include identifiers to make explicit that multiple records derive from a single standardised sample (vegetation plot survey, bird count, eDNA sample etc.) or relate to a single individual of the species (animal tracking). Field observation records are a fundamental unit for considering past and present species distributions, spatial occupancy, community composition, ecosystem health etc. Many of the Essential Biodiversity Variables (EBVs, Pereira et al. 2013, Kissling et al. 2018, Hardisty et al. 2018, Hardisty et al. 2019) can be constructed using such records. Each field observation record also serves as a pointer to the varied and potentially much richer information that may be associated with a field study (methods and protocols, community composition, associated environmental measurements etc.), a citizen science record (images, sound recordings, notes), eDNA (sequences, community composition), animal tracking (time series of locations for the same individual) or any other suitable source of evidence.

Sampling event data are currently only handled as a source of occurrence records, although Darwin Core elements, such as eventID and samplingProtocol, are retained with each record, allowing users of the data to make use of the associated information. At present, relatively few datasets are published to the ALA using the Darwin Core standard event class (https://dwc.tdwg.org/terms/#event), and the ALA currently handles these data only as a source of occurrence records. It would be relatively simple, however, for holders of survey and monitoring data (e.g. vegetation plot data, standardised bird and frog counts, pitfall and Malaise sample data, environmental DNA sequencing) to share their data in this form. All such data can continue to contribute to the ALA occurrence record index – the Biocache – but can also contribute to future data products that enable researchers to locate and compare sites across space and time. The greater standardisation associated with these data may also assist with validation and calibration of other ALA data.

#### Animal tracking data

With increasing miniaturisation and lowering costs, tracking devices are more commonly being attached to animals to monitor their movements. The data these devices supply offer rich insights into the habits of the organisms and their use of the landscape. For migratory species, they are a key tool to identify key resources along their routes. A ping from an electronic tag on an animal is, in Darwin Core terms, a ‘machine observation’. Tracking data for a single animal may be very detailed, organised as a series of coordinates and timestamps.

The ALA inherited an animal tracking software that was originally called OzTrack (Dwyer et al. 2015) and is now called ZoaTrack (Newman et al. 2020, https://zoatrack.org/). This is a portal that covers import of track data, storage, visualisation, analysis and export. Supported spatial analysis methods with ZoaTrack include the minimum convex polygon, kernel utilisation distribution, kernel Brownian bridge, alpha hull, local convex hull, heat map (point intensity), heat map (line intensity) and Kalman filter. Movement metrics include the total number of locations for that animal, the mean number of detections per day, the maximum number of detections per day, the distance moved along the track using great circles (km), the mean step length (km) and the mean step speed (km/h). The results can be exported as KML files or shapefiles.

Animal tracking data are imported from ZoaTrack into the Biocache in a minimal form. The Biodiversity Information Standards Interest Group on Machine Observations (https://www.tdwg.org/community/mobs/) has been working on two standards for these data, one for encoding the tag data directly and one that exposes these data using the Darwin Core standard. Currently, tracks are imported as occurrence records in what are best described as master records: one record per track. The convex hull of the track is calculated and added to the Darwin Core term dwc:footprintWKT, and other information, such as dwc:scientificName, dwc: eventDate (as a range) and, in effect, metadata, is added to the relevant Darwin Core terms. This method was used to enable tracks to be used with any other records of the species, to be displayed as a convex hull and detected as intersecting an area of interest in the Spatial Portal’s area reports. This strategy has minimal performance impact on the ALA occurrence record store, the Biocache, and still provides direct access from the master record to all associated data in ZoaTrack.

### Collections and data resources

**Figure 5.**
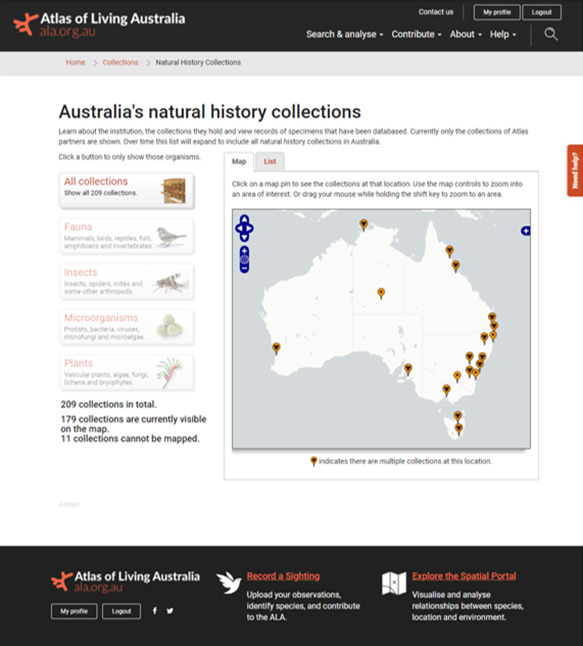
Australia’s natural history collections. This page provides a way for users to investigate data from museums, herbaria and collections in universities (https://collections.ala.org.au/).

Information about Australia’s curated natural history collections can be explored in the ALA through the Natural History Collections page (https://collections.ala.org.au/) (Fig. 5). There are 209 collections (at October 2020) and several collections may be associated with a single institution (often organised around taxonomic groups). Museums, herbaria, university collections and CSIRO’s national collections usually provide the Darwin Core elements dwc:institutionCode and dwc:collectionCode as a part of their specimen data records, reinforcing the linkage between specimen data and collection information.

Data within the ALA are also organised according to Data Resource (or dataset) and Data Partner. There are 8160 Data Resources in the ALA (https://collections.ala.org.au/public/datasets) and these are grouped under Data Partners. Australia’s major museums and CSIRO national collections, for example, all contain the phrase ‘provider for OZCAM’ in their data resource name. This reflects ALA history, where the original datasets that formed the basis of the data in the ALA came from the museums data aggregator as the Data Partner – the Online Zoological Collections of Australian Museums (OZCAM). Similarly, herbaria have ‘AVH’ in their data resource name, reflecting the data aggregator for the herbaria – the Australasian Virtual Herbarium (AVH).

### Biodiversity literature

The Biodiversity Heritage Library (BHL) is a global consortium of natural history, botanical and research libraries based in museums, herbaria and universities, plus national and state libraries. The mission of the BHL is to digitise literature related to biodiversity and natural history and to make it freely and openly accessible. The secretariat for the consortium is based at the Smithsonian Libraries in Washington, D.C. and the global website is managed through that body. More than 58 million pages of scientific literature, with content ranging from the 15^th^ century to the present day, can be searched through the website (https://www.biodiversitylibrary.org/).

In partnership with the ALA, the Biodiversity Heritage Library project in Australia was started in 2010. It was originally established to provide a literature service to support taxonomic names within ALA. Within the ALA website, access to the content in BHL is available from the Literature tab on species pages.

The project is based at Museums Victoria in Melbourne, where the digitisation equipment is held. A small team of paid staff work collaboratively with Museums Victoria librarians to nominate, locate and make available rare books, monographs and serials for scanning. Since the project first started, much of the digitisation and post-processing has been done by an active and extremely committed team of volunteers, some of whom have been with the project since its inception. This small team has digitised more than 350,000 pages of literature over the course of the last decade, and has focused more recently on literature published in Australia.

In recent years, the BHL Australia project has invited participation from museums, herbaria, learned societies, field naturalist groups and other organisations that produce publications, but that may not have the means to digitise their content. Thirty organisations have so far contributed publications to BHL via the Australian project (https://www.biodiversitylibrary.org/collection/bhlau). The Australian team have also worked with project leaders in New Zealand to initiate a similar project there.

Future possibilities exist to use the published literature as a source of taxonomic names and of occurrence records. This may be developed further in the next few years.

### Species lists

Within the ALA, lists of species names can be used to link multiple taxa into a group with a common characteristic. The types of lists that are supported include:

- taxonomic checklists that include names and classifications for all species within a group (either at the global scale or for a defined region)
- local occurrence checklists that list all species recorded from an area (e.g. a national park)
- lists of species with a common conservation status
- lists of species with a common introduced or invasive status
- lists that summarise diagnostic, morphological, ecological or distribution information for each listed species (a simple form of species profile data).

At the most basic level, lists in the ALA are defined as a set of two or more taxon names (at any taxonomic level), usually supplied as a CSV-formatted file or cut/pasted into the Lists portal (https://lists.ala.org.au). As with most information in the ALA, lists can be created by ALA-registered users and, if not designated as ‘private’, are publicly discoverable and usable. More than 5000 lists have been created by research scientists, taxonomists, citizen scientists, area managers, environmental consultants and members of the public. During 2020, there were more than 5000 users of the Lists tool.

Several tags can be associated with each list:

- authoritative (created by acknowledged responsible agency, committee or expert)
- included in species pages (referenced on the species summary pages)
- private (only visible to the user who uploaded the list)
- invasive species (species with any invasive status anywhere in the Australian region)
- member of Sensitive Data Service (species have authoritative sensitivities)
- region provided (a formal area for the list has been defined)
- threatened species list (species with any conservation status in Australia).

Species character lists provide for any number of traits or attributes to be associated with each name in the list. This information is currently unconstrained, meaning that trait labels are local to each list, rather than being consistently interpreted across lists. Traits can take any text or numeric value. Future developments could use accepted vocabularies, thesauri and ontologies to constrain trait labels and states or expected value ranges. A simpler version of this is done for the ALA Sandbox portal (https://sandbox.ala.org.au), which attempts to match occurrence record header terms against the Darwin Core standard. This strategy would provide a practical impetus for an evolution to international standards and would enable the Lists portal to detect trait types and values, and to flag exceptions.

Authoritative lists are those for which a recognised expert, institution or committee has formal responsibility. For example, the Environment Protection and Biodiversity Conservation Act, managed by the Australian Department of Agriculture, Water and the Environment (https://www.environment.gov.au/epbc/about), lists species that are ‘extinct, extinct in the wild, critically endangered, endangered, vulnerable or conservation dependent’. The ‘Authoritative’ tag is applied only by the list administrators after consultation with the authority.

### Biodiversity projects

The ALA accommodates information on biodiversity projects – for example, from government-funded on-ground interventions, such as weed management and riparian re-vegetation activities, or from projects conducted by research and citizen science sectors. In both cases, the ALA facilitates project documentation. MERIT (https://fieldcapture.ala.org.au) is a platform for government-mandated planning and reporting. The ALA BioCollect platform (https://biocollect.ala.org.au) supports biodiversity-related projects by capturing both descriptive metadata for each project and raw primary field data. BioCollect has a strong citizen science emphasis.

The ALA provides these capabilities for the collection of observational data as they complement data from other sources and focus on data collection from systematic surveys. For example, projects dealing with the management of alien invasive species may be added to the ALA’s list of records and, as with all occurrence records, are then available for viewing, analyses and downloading. The intent is to facilitate the discovery of relevant on-ground projects when examining area reports from MERIT, BioCollect and the ALA’s Spatial Portal.

Projects registered into these ALA systems fall into three categories. At present, these categories are not used as filters elsewhere in the ALA:

- ecological science (1600-plus projects at https://www.ala.org.au/biocollect-for-ecosciences/)
- natural resource management activities (15 projects at https://www.ala.org.au/biocollect-for-natural-resource-management/)
- citizen science (524 projects at https://www.ala.org.au/biocollect-for-citizen-science/)

Ecoscience projects (https://biocollect.ala.org.au/ecoscience) are generally not open for public participation and focus on environmental assessment and monitoring surveys. These are often, but not exclusively, established by scientists collecting data for their own research projects or by ecologists and natural resource management practitioners undertaking surveys for planning-related development applications and long-term site monitoring projects.

Natural resource management (NRM) projects support environmental NGOs, NRM organisations, community groups and local governments to create, manage and record data and communicate project outputs and outcomes to their communities (see https://www.ala.org.au/biocollect-for-natural-resource-management/). These projects record the sequence of activities which are undertaken to restore, re-vegetate and rehabilitate environmental areas over a period. Many of these activities include observation, collection, monitoring and establishment of plants and/or animals. In addition to any occurrence records shared this way, the associated project, survey and activity information can also be extremely useful to the broader scientific community in providing context and descriptive information to interpret records. For example, information about re-vegetation, site restoration, seed collection, weed and pest management are fundamental to understanding ecological processes in an area or region.

Citizen science projects (https://www.ala.org.au/biocollect-for-citizen-science/) are open to involvement by anyone and BioCollect contributes to community outreach for project support. Most of the citizen science projects relate to environmental observations but, as BioCollect is a platform that supports international citizen science projects and is closely aligned with the Australian Citizen Science Association (ACSA, https://citizenscience.org.au/), projects can be of any nature. These citizen science projects can also be accessed via the ACSA website (https://citizenscience.org.au/ala-project-finder/) or through global access to their metadata (PPSR Core metadata, https://github.com/CitSciAssoc/DMWG-PPSR-Core/wiki/About-the-PPSR-Core).

### Environmental layers

Experiences in managing the ALA environmental data have largely been captured by Belbin and Williams (Belbin and Williams 2015), so this paper provides a brief summary of data, services and the lessons learnt. A recent review of environmental layers across four Australian biodiversity infrastructures can also be viewed at https://support.ecocloud.org.au/support/solutions/articles/6000237060-key-environmental-data-layers-across-ecoscience-facilities.

When the ALA received expanded funding in 2009, it recruited an analytical ecologist to support ecological and related research. A cross-section of experienced ecological scientists were invited to two workshops. The first of these (Flemons et al. 2009) evaluated potential tools and methods that the ALA could support, and the second (Flemons and Belbin 2010) identified what data were required to support those tools. It was readily apparent that the ALA needed to integrate a wide range of environmental data if it were to support ecological research. The ALA references these datasets as ‘environmental layers’ to distinguish them from the point-based species occurrence data that form most of the ALA’s data holdings.

When the ALA was established, no other Australian agency held more than 10% of the layers necessary to support research and environmental management. The environmental layers currently held by the ALA can be viewed at https://spatial.ala.org.au/layers. A three-level classification scheme was developed to help discovery of relevant layers in the ALA. Layers may be gridded datasets with continuous values representing variables, such as mean annual temperature or soil pH. These layers have been selected based on the potential of the measured variables to influence the spatial distribution of species. The ALA also includes datasets identified as ‘contextual layers’. These generally have a polygonal structure and belonged to defined classes, such as states and territories or bioregions. Environmental layers provide context for understanding the management of Australian species. They are classified into major categories: area management, biodiversity, climate, distances, fire, hydrology, land management, marine, political, sensitive data, social, substrate, topography and vegetation. Each month, four global coverage internally generated layers are created from all occurrence records: species occurrence density, species richness, endemicity and Shannon Diversity (H).

Contextual layers are also cross-tabulated with all the ALA’s occurrence records. For example, cross-tabulating the Australian states and territories layer with the terrestrial CAPAD (parks and reserves) layer reports the area in square kilometres, the number of taxa and the number of occurrence records in each of the areas defined by the spatial intersection of the two layers. As the occurrence records are added to on an *ad hoc* basis, the cross-tabulations for species and occurrences need to be updated regularly for all pairs of contextual layers. This process imposes a significant load on ALA computers, but enriches the data available to users.

The ALA's interest in these environmental layers is secondary to its primary purpose of documenting species. They are, however, a valuable source of environmental properties that the ALA uses to enrich all occurrence data, and they contribute to visualisation and analysis tools in the Spatial Portal and other ALA portals.

### Descriptive species information

The ALA Biodiversity Information Explorer or BIE (https://bie.ala.org.au) offers species pages that serve as a standard entry-point for those exploring occurrence records. The home page search links users to the BIE species pages.

Species pages in the ALA provide access to aggregated descriptive information about species. Common to all pages is an overview, containing:

- descriptive text, which may have been obtained through third-party APIs
- threatened species or conservation status, derived from state, territory and federal threatened species lists
- images, aggregated from media uploaded along with occurrence data
- taxonomic names and synonyms, brought in from the source names list
- classification hierarchy, also brought in from the source names lists
- relevant literature, sourced by sending queries on the species name to the Biodiversity Heritage Library and to Trove, run by the National Library of Australia
- Links to genetic data using the GenBank API
- a list of Data Resources (and Data Partners if relevant) that have supplied occurrence records

Several recent projects have broadened the authored informative content in the ALA. A noteworthy example is the Indigenous Ecological Knowledge project. In this project, senior knowledge holders from several Aboriginal communities have worked with non-Aboriginal scientists and linguists to document local names for plants and animals. The ALA website includes names from the Kamilaroi/Gamilaroi and Yuwaalawaay/Yuwaalayaay languages for over 250 species. The Indigenous Ecological Knowledge project continues to work actively with senior knowledge holders in various communities around Australia. Common to all Indigenous Ecological Knowledge projects is the aim to bridge the gap between traditional and non-Aboriginal science knowledge by working on country with communities who want to share traditional knowledge in a culturally safe way. The next phase of the Indigenous Ecological Knowledge project will use the species descriptive authoring tool (Profiles: https://profiles.ala.org.au) to record information about the descriptions and concepts embodied in the names. The Profiles platform is used for the Flora of Australia project (https://profiles.ala.org.au/opus/foa). This project contains authored content that describes plants using technical language and is mainly used by botanists to aid in descriptions and identifications.

## Gaps in data and services

As new features are added, the complexity of the ALA increases. While improved functionality is often valued by the users, the downside is increased computational and human data-processing overheads. In contrast to some biodiversity data infrastructures, the data types accepted by the ALA are wide-ranging, and the tools provided in the ALA support an unusually wide range of services and users. A positive outcome of this is that the ALA supports a diverse range of communities. The challenges, however, include the number of applications requiring ALA technical support, a lack of consistency in user interfaces and code base, challenges associated with generating consistent and comprehensive documentation, and the complexity associated with integrating and linking data.

One of the more significant gaps is the need for more comprehensive standards and better attention by data providers to compliance with those standards already in use. Darwin Core (https://dwc.tdwg.org/) is a fundamental standard used by the ALA and related infrastructures. It provides the elements needed to allow any observation of a species or occurrence record for a specimen to be aligned into an occurrence database. This, in turn, enables records of species to be searched by, or filtered for, indexed Darwin Core terms, such as species name, location or date of observation. Darwin Core has been developed as a very flexible standard that can support the widest possible range of data sources sharing spatiotemporal data on species occurrence, but this flexibility is achieved by placing only minimal semantic constraints on published data. Different data publishers may adopt different strategies for mapping data from otherwise comparable specimens or field surveys into Darwin Core and this may lead to inconsistencies in how the data are subsequently interpreted or used. Data in the ALA, moreover, could be improved. An example is in the low use of dwc:samplingProtocol. Approximately 98.5% of the occurrence records in the ALA have a dwc:occurrenceStatus of ‘present’, while the remainder are listed as ‘absent’ or ‘unknown’. This would be typical of most infrastructures. What is hidden, however, is the many absences or ‘unknowns’ that could be determined from another Darwin Core element: dwc:samplingProtocol. Unfortunately, dwc:samplingProtocol has been provided for only 0.4% of the ALA’s records, even though we know that a far larger proportion of records could have this element filled, as they are the result of systematic sampling but are not recorded as such. This includes most of the records from Birdlife Australia and eBird.

While most biodiversity data infrastructures have adopted the Darwin Core standard, a recent study on the production of Essential Biodiversity Variable (Hardisty et al. 2019) highlighted the challenge of trying to merge GBIF and ALA records of three species. Records in GBIF and the ALA are processed differently, resulting in inconsistent use of terms derived from the raw or verbatim data. The study also showed that there were Australian records in GBIF that were not in the ALA and vice versa. A current project has begun to align GBIF and ALA occurrence record processing infrastructure to achieve greater alignment and efficiencies.

The ALA has several other data streams for which necessary standards are not well established to structure the data. Examples include trait data, annotations on occurrence records, occurrence record tests and bioenvironmental data classification. This lack of standards is not unique to the ALA, but it does present significant barriers to efficient development for the acquisition, processing and sharing of biodiversity-related data and services. The development of standards is complex and time-consuming and there is no guarantee that, once work is complete, proposed standards will be adopted. The ALA, however, does invest considerable time to contribute to internationals standards development. It does this through its involvement over many years in the Biodiversity Information Standards (TDWG) organisation. The ALA is committed to continuing to invest in efforts to develop biodiversity data standards given how critical they are in supporting data integration, nationally and internationally.

## Major international activities

The ALA’s role in hosting the Australian node of the Global Biodiversity Information Facility (GBIF) is one of its most important partnerships. Since its inception, the ALA has worked closely with GBIF to ensure occurrence records in the Australian region and beyond are supplied to GBIF. Occurrence records relevant to the Australian region and published to GBIF are also imported into the ALA.

GBIF catalyses a great deal of international activity around biodiversity data mobilisation, standardisation and use, and actively promotes associated training and capacity enhancement efforts around the world. This has included the development of the Global Biodiversity Informatics Outlook (Hobern et al. 2012) and the *alliance for biodiversity knowledge* (Hobern et al. 2019). The *alliance* is a consortium-based approach to increasing the cooperation and synergy between biodiversity information producers, managers and users to maximise benefit for all. The ALA's various contributions to international biodiversity fit well into this approach, with multiple innovations and tools now widely used in national and regional portals around the world.

Biodiversity Information Standards (TDWG) provides one of the most significant foundations for ALA activities. The ALA’s dependence on biodiversity informatics standards provides a strong case for the practical support of TDWG. This is done through ALA staff contributing time to current standards developments such as Data Quality, Audubon Core, Citizen Science metadata and others.

Over the past seven years, a community of national atlases, based on the ALA’s open-source code and supported by GBIF, has also been developed (see https://living-atlases.gbif.org/). Since 2013, the Living Atlases community has organised technical workshops to present ALA modules to other institutions, to improve already existing national data portals and to learn from each other’s achievements. As at March 2021, 27 Living Atlas sites are online, three are under development and seven more are being explored, highlighting the global impact of the ALA and NCRIS programmes.

## ALA future directions

As the ALA entered its 10th year in production in 2020, it undertook a Future Directions National Consultation process to provide information for its forward strategy. Consultation began in early June 2019 with the design of the interview approach and development of a stakeholder engagement plan framed around five key user domains: research, government, industry, community and international. Stakeholders were partitioned into three types:

- data contributors
- ALA users
- those with an institutional interest in the operation of the ALA.

The process adopted a semi-structured interview approach framed around a strengths, weaknesses, opportunities and threats (SWOT) framework (Table 2). It captured feedback from more than 100 Australian and international stakeholders and 35 organisations.

Results highlighted that the ALA has pioneered a step-change in the way that Australia’s biodiversity data are utilised through its approach to open data access, by providing innovative products and services and mobilising the national biodiversity community. The ALA’s global impact received recognition through both its technical and strategic contribution to the Global Biodiversity Information Facility (GBIF) and its technical leadership of the global Living Atlases initiative. In this regard, the ALA was acknowledged as one of the world’s foremost national biodiversity data infrastructures. The consultation also highlighted that the expectations of the ALA’s stakeholders are rapidly evolving, and that the ALA has not always adapted as well as it might have. Notable concerns to address if the ALA is to realise its full potential as an operational data infrastructure include:

- data quality - this is critical for reliable decision-making and quality research outputs. It is also important if the ALA seeks to move, not only to aggregate data, but also to have a custodial role in curating data.
- data diversity - this is needed to ensure that the ALA can effectively deliver to major national biodiversity reporting, assessment and monitoring programmes and help to address the ‘big questions’ in biodiversity research. It requires datasets that are more diverse, representative or comprehensive in terms of geography, time and taxonomy. The ALA will also need to assist partners to prioritise data collection and digitisation efforts to align with national needs.

With respect to opportunities, stakeholders encouraged the ALA to:

- include more industry data as, in some jurisdictions, this can represent more than 85% of the current biodiversity survey effort and would provide a useful complement to the data already harmonised or potentially available from museums, collections and state biodiversity data programmes
- focus the ALA’s product and services portfolio to reduce some confusion about the mission and scope of the ALA and to provide ongoing support for priority operations
- support national capability to provide standard, interoperable biodiversity data by assisting state and territory biodiversity data systems to align their approaches with respect to standards and with hard and soft data infrastructure.

The ask from stakeholders from a national biodiversity infrastructure was clear. Stakeholders want:

- data of high quality that have been corrected for obvious errors and include metadata that indicate fitness for purpose, including suitability for research
- relevant data from a range of users that address key biodiversity questions, including longitudinal data, data of comprehensive geographic and wide taxonomic spread and monitoring data from government programmes, consultants and industry
- integration of new data streams beyond occurrence records, such as images, genomics, sound recordings and environmental assessment data
- tools and standards to assist in the collection, integration, analysis and synthesis of these data.

It was also clear that stakeholders saw a key role for the ALA in ‘soft diplomacy’ – to be a catalyst, to offer leadership by facilitating cooperation and collaboration between institutions. The ALA has an ongoing role in identifying gaps in the national capability and working with others to ensure that these are filled, even if not by the ALA itself. The ALA’s relationships with other NCRIS facilities and international bodies were also seen to be an important way of fulfilling this part of its role. The ALA is well positioned to shape a future role that continues to be critical in the delivery of national biodiversity data. The recently released ALA Strategy 2020–2025 further articulates these strategic priorities (https://www.ala.org.au/publications/, last accessed 13 October 2020).

In response to these findings, the ALA has recently embarked on a number of transformative projects through its annual work planning process. This includes the ALA data quality project which culminated in the recent release of the data profiles functionality (https://www.ala.org.au/data-quality-project/) and a core infrastructure upgrade project that is being delivered in close partnership with GBIF and will improve alignment between ALA and GBIF systems and data. Work in 2020-2021 includes investment in an ALA complex data project to support richer data types than can currently be captured as Darwin Core occurrence records and the beginnings of an industry engagement programme to identify the ability of this sector to contribute data to the ALA. The ALA will also be working more closely with partners in the Integrated Marine Observing System, Terrestrial Ecosystem Research Network and the Australian Research Data Commons to develop cross-facility data assets to support national environmental reporting (https://ardc.edu.au/project/ecoassets/). This is in response to recommendations that the ALA should expand its partnerships with related facilities to deliver greater collective benefit to users. Further detail of ALA's plans may be found in the ALA Strategy 2020-2025 and the ALA Annual Work Plan 2020-2021. These plans, along with future annual work plans as they are published, may be accessed online at https://www.ala.org.au/publications/.

## Conclusion

It has been 10 years since Australia’s museums and herbaria were the key force in encouraging the establishment of the Atlas of Living Australia under Australia’s National Collaborative Research Infrastructure Strategy (NCRIS). In that decade, more than 90 million occurrence records of 110,000 species, 5000 species lists, 2000 biodiversity-related projects, 500 environmental layers, scores of portals and the underlying code have been made openly available. Further, the establishment of 24 national Living Atlases based on the ALA architecture attest to its success in delivering biodiversity data and related services. In turn, these services and capabilities support research excellence in fields such as biodiversity, genetics and ecosystem science; they support major government and community-led natural resource management programmes; and, through the ALA’s close relationship with the Global Biodiversity Information Facility, Australian biodiversity data are made available to the world. These achievements have been enabled by strong partnerships and contributions from multiple stakeholders in Australia to build what remains one of the world's foremost biodiversity data infrastructures.

## Glossary

- ABCD: Access to Biological Collections Data, a TDWG standard
- ABRS: Australian Biological Resources Study, part of the Australian Department of Agriculture, Water and the Environment
- ALA: Atlas of Living Australia, an NCRIS facility
- ALA4R: The ALA’s R-based library of functions
- API: Application Programming Interface
- APNI: Australian Plant Name Index
- ARCS: Australian Research Collaboration Service, the original name for the ARDC
- ARDC: Australian Research Data Common, an NCRIS facility
- AURIN: Australian Urban Research Infrastructure Network, an NCRIS facility
- AVH: Australian Virtual Herbarium
- BHL: Biodiversity Heritage Library
- BIE: Biodiversity Information Explorer
- Biocache: The ALA’s occurrence database
- BioCollect: The ALA’s project-based portal
- BPA: BioPlatforms Australia, an NCRIS facility for genomics, proteomics, metabolomics and bioinformatics
- CAPAD: Collaborative Australian Protected Area Database, a database listing all Australian biologically-related parks and reserves collated by the Australian Department of Agriculture, Water and the Environment
- CHAH: Commonwealth Heads of Herbaria
- CHAFC: Commonwealth Heads of Faunal Collections
- CSIRO: Commonwealth Scientific and Industrial Research Organisation
- DigiVol: The ALA’s citizen science volunteer portal
- Diversity: In the context of this paper, the number of different species
- DNA: Deoxyribonucleic acid, a self-replicating material which is present in nearly all living organisms as the main constituent of chromosomes. It is the carrier of genetic information.
- eDNA: Environmental DNA, DNA found within samples of the environment (e.g. in soil or water)
- Endemism: A taxon that is localised to a location – it does not occur outside that location
- ERIN: Environmental Resources Information Network (a previous project with the Australian Department of Agriculture, Water and the Environment)
- GBIF: The Global Biodiversity Information Facility
- GBIO: Global Biodiversity Information Outlook
- HISPID: Herbarium Information Standards and Protocols for Interchange of Data, a TDWG standard
- IDigBio: Integrated Digitized Biocollections, the USA facility for digitisation of biodiversity collections, funded by the National Science Foundation
- IMOS: Integrating Marine Observing System, an NCRIS facility
- IPBES: The Intergovernmental Science-Policy Platform on Biodiversity and Ecosystem Services
- MERIT: Online reporting tool for projects supported by the Department of Agriculture, Water and the Environment
- NCRIS: National Collaborative Research Infrastructure Strategy
- NSL: National Species List
- Occurrences: Observations of species or specimen records that generally use the Darwin Core standard
- OZCAM: Online Zoological Collections of Australian Museums
- Specimen: A physical instance of a species
- Synonym: An alternative name for a taxon
- Taxon/taxa: A group of one or more populations of an organism or organisms, seen by taxonomists to form a unit
- Taxonomy: A branch of science that encompasses the description, identification, nomenclature and classification of organisms
- TCS: Taxonomic Concept Schema, a TDWG standard
- TDWG: Taxonomic Database Working Group, now better described as Biodiversity Information Standards
- TERN: Terrestrial Ecosystem Research Network, an NCRIS facility
- Trait: An attribute of a specimen, species or group of species
- UNESCO: The United Nations Educational, Scientific and Cultural Organization
- ZoaTrack: The ALA portal for import, analyses and export of animal track data (formerly OzTrack)

## Figures and Tables

**Table 1.** Major ALA data types and URLs for public access.

**Data**	**URL**
Species occurrence data	https://biocache.ala.org.au
Animal tracking data	https://zoatrack.org/
Specimen data	https://specimens.ala.org.au/
Natural history collections	https://collections.ala.org.au/
Biodiversity literature	https://www.biodiversitylibrary.org/collection/bhlau
Species lists	https://lists.ala.org.au
Biodiversity projects	https://biocollect.ala.org.au
Environmental layers	https://spatial.ala.org.au/layers
Descriptive species information	https://bie.ala.org.au/

**Table 2.** Summary of ALA Future Directions National Consultation against a strengths, weakness, opportunities and threats framework.

**Strengths**	Software team of high calibre and critical mass that solves the complex data interoperability issues to harmonise biodiversity data. Impressive amount of Australia's biodiversity data, particularly plant and bird data which are of good quality and can be accessed for free. User-friendly interface and good IT products that have underpinned and improved national and global awareness of, and access to, Australia's biodiversity holdings/collections. Well networked and well regarded domestically and internationally and has built a national community that is working to improve provision of biodiversity data. Strong institutional support from CSIRO that has helped the ALA to weather funding uncertainty and to retain its quality staff.
**Weaknesses**	Lack of clear strategy and priorities for developing the work programme and lack of consultation about this in the past. Too many disconnected products and services because the work programme is driven by project funding and opportunity rather than by a focused strategy. Data quality or fitness for purpose can be hard to assess and poor in some cases, including reliability of taxonomic names, lack of absence data or information about the quality of species identifications. Data types are not comprehensive; for example, the ALA lacks genomics data and longitudinal (i.e. survey) data at scale or from a national perspective. Data are not targeted to key national biodiversity questions or assessments, but rather may reflect historical inconsistency of past sampling strategies, given the initial focus on collections and museum data.
**Opportunities**	Provide national leadership and coordination with respect to standards, biodiversity informatics, data quality and future system development. Deliver a more integrated national data capability and suite of services through partnerships with related NCRIS facilities. Become a data repository for monitoring surveys and environmental assessments collected by government and industry. Collect and digitise data that address key biodiversity-related research questions. Provide analytics that can support decision-making or research insights, including in new areas, such as biosecurity.
**Threats**	Lack of ongoing resources because of dependence on government funding. Reputational risk through poor data quality or failure to engage more with subject matter experts in taxonomy and ecological sciences. Unclear mandate undermined by competitors who can better deliver specialised portals at lower cost. Failure to deal with new data streams in ecology and genomics, including the variety, volume and velocity of data flow that will be difficult to integrate. Owners of data not willing to share data openly and nationally due to the constraints they work in.

## Hosting institution

Commonwealth Scientific and Industrial Research Organisation
